# Thermoelectricity in molecular junctions with harmonic and anharmonic modes

**DOI:** 10.3762/bjnano.6.218

**Published:** 2015-11-11

**Authors:** Bijay Kumar Agarwalla, Jian-Hua Jiang, Dvira Segal

**Affiliations:** 1 Chemical Physics Theory Group, Department of Chemistry, and Centre for Quantum Information and Quantum Control, University of Toronto, 80 Saint George St., Toronto, Ontario, M5S 3H6, Canada; 2 Department of Physics, Soochow University, 1 Shizi Street, Suzhou 215006, China

**Keywords:** counting statistics, efficiency, molecular junctions, quantum transport, thermoelectricity

## Abstract

We study charge and energy transfer in two-site molecular electronic junctions in which electron transport is assisted by a vibrational mode. To understand the role of mode harmonicity/anharmonicity in transport behavior, we consider two limiting situations: (i) the mode is assumed harmonic, (ii) we truncate the mode spectrum to include only two levels, to represent an anharmonic mode. Based on the cumulant generating functions of the models, we analyze the linear-response and nonlinear performance of these junctions and demonstrate that while the electrical and thermal conductances are sensitive to whether the mode is harmonic/anharmonic, the Seebeck coefficient, the thermoelectric figure-of-merit, and the thermoelectric efficiency beyond linear response, conceal this information.

## Introduction

Molecular electronic junctions offer a rich playground for exploring basic and practical questions in quantum transport, such as the interplay between electronic and nuclear dynamics in nonequilibrium situations. Theoretical and computational efforts based on minimal model Hamiltonians are largely focused on the Anderson impurity dot model which consists a single molecular electronic orbital directly coupled to biased metal leads, as well as to a particular vibrational mode [1]. Since the same molecular orbital is assumed to extend both contacts, the model allows for simulations of transport characteristics in conjugated molecular junctions with delocalized electrons.

**Figure 1:**
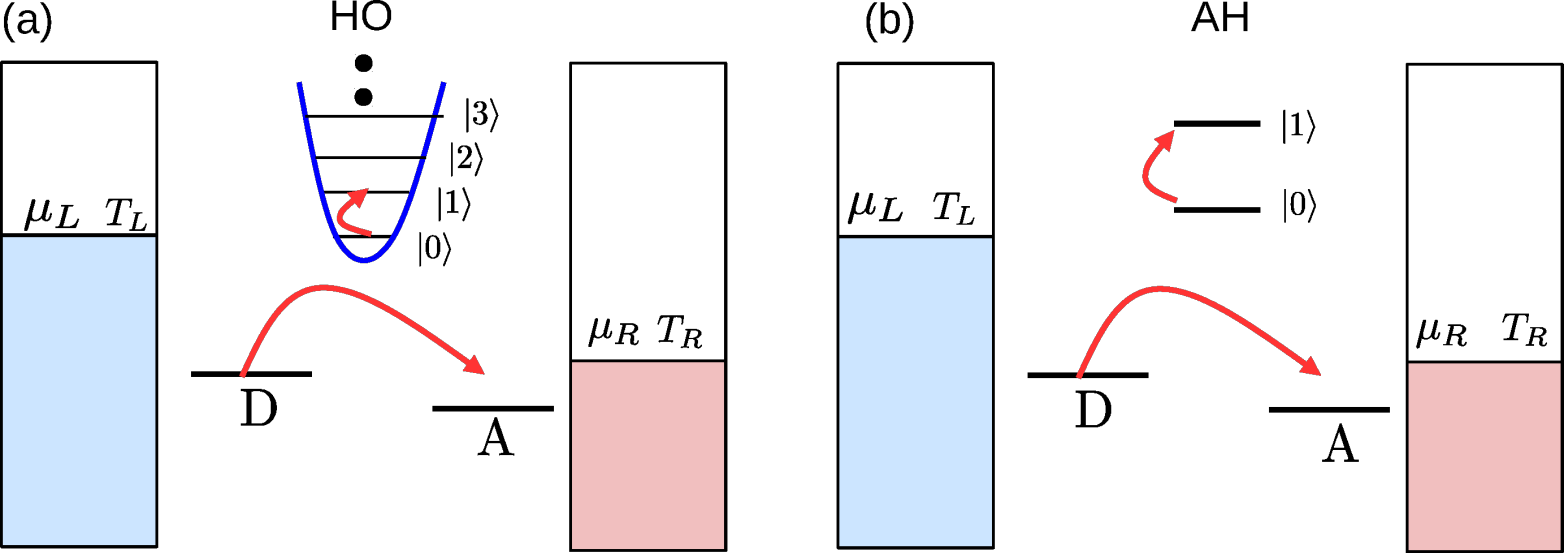
Scheme of the D–A model considered in this work. A two-site (D, A) electronic junction is coupled to either (a) a harmonic molecular mode, or (b) an anharmonic mode, represented by a two-state system. The molecular mode may further relax its energy to a phononic thermal reservoir (not depicted here), maintained at temperature *T_ph_*.

In this work, we focus on a different class of molecular junctions as depicted in Figure 1. In such systems, two electronic levels are coupled via a weak tunneling element, but electrons may effectively hop between these electronic states when interacting with a vibrational mode. This could e.g., correspond to a torsional motion bringing orthogonal π systems into an overlap as in the 2,2’-dimethylbiphenyl (DMBP) molecule recently examined in [2–4].

This model is related to the original Aviram–Ratner construction for a donor–acceptor molecular rectifier [5]. Thus, we identify the two states here as “D” and “A”, see Figure 1, and refer to the model as the “D–A junction”. More recently, this construction was employed for exploring vibrational heating and instability under a large bias voltage [6–8]. The system is also referred to as the “dimer molecular junction” [9], or an “open spin–boson model” [10] (where the spin here represents the D and A states, the bosons correspond to the molecular vibrational modes, and the system is open, i.e., coupled to metal leads). It was utilized to study charge transfer in donor–bridge–acceptor organic molecules [11] and organic molecular semiconductors [12], as well as thermoelectric effects in quantum dot devices [13–14].

Recently, Erpenbeck et al. had provided a thorough computational study of transport characteristics with nondiagonal (or nonlocal) as well as diagonal (local) electron–vibration interactions [15]. Here, in contrast, we simplify the junction model and omit the contribution of direct tunneling between the D and A units. This simplification allows us to derive a closed (perturbative) expression for the cumulant generating function (CGF) of the model, which contains comprehensive information over transport characteristics.

Measurements of charge current and electrical conductance in single molecules hand over detailed energetic and dynamical information [16]. Complementing electrical conductance measurements, the thermopower, a linear response quantity, also referred to as the Seebeck coefficient, is utilized as an independent tool for probing the energetics of molecular junctions [17–24]. Experimental efforts identified orbital hybridization, contact-molecule energy coupling and geometry, and whether the conductance is HOMO or LUMO dominated. More generally, the thermoelectric performance beyond linear response is of interest, with the two metal leads maintained at (largely) different temperatures and chemical potentials.

What information can linear and nonlinear thermoelectric transport coefficients reveal on molecular junctions? Specifically, can they expose the underlying electron–phonon interactions and the characteristics of the vibrational modes participating in the process? Focusing on the challenge of efficient thermoelectric systems, how should we tune molecular parameters to improve heat to work conversion efficiency? These questions were examined in recent studies, a non-exhaustive list includes [13–14,25–36].

We focus here on the effect of vibrational anharmonicity on thermoelectric transport within the D–A model. To explore this issue, two limiting variants of the basic construction are examined, as displayed in Figure 1: (a) The vibration is harmonic in the so-called “harmonic oscillator” (HO) model. (b) To learn about deviations from the harmonic picture, we truncate the vibrational spectrum to include only its two lowest levels, constructing the “anharmonic” (AH) mode model. In a different context, the AH model could represent transport through molecular magnets, in which electron transfer is controlled by a spin impurity [37].

The complete information over transport behavior is contained in the respective cumulant generating functions, which we provide here for the HO and the AH models, valid under the approximation of weak electron–vibration interactions. From the CGFs, we derive expressions for charge and heat currents, and study the linear and nonlinear thermoelectric performance of the junctions. Focusing on the role of vibrational anharmonicity, we find that while it significantly influences the electrical and thermal conductances, nevertheless in the present model it does not affect heat-to-work conversion efficiency.

## Model

We consider a two-site junction, where electron hopping between the D and A electronic states (creation operators 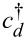 and 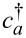, respectively) is assisted by a vibrational mode. The total Hamiltonian is written as

[1]
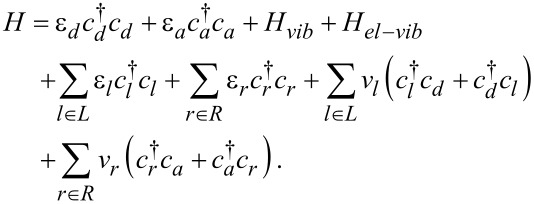


The molecular electronic states of energies ε*_d,a_* are hybridized with their adjacent metals, collection of noninteracting electrons, by hopping elements *v_l_* and *v_r_*. Here 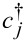 (*c_j_*) is a fermionic creation (annihilation) operator. The electronic Hamiltonian (Equation 1, excluding *H_vib_*+ *H_el−vib_*) can be diagonalized and expressed in terms of new fermionic operators *a_l_* and *a_r_*,

[2]
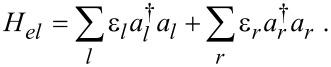


The molecular operators can be expanded in the new basis as,


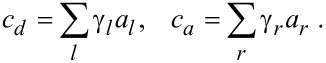


The γ*_l,r_* coefficients satisfy [7]


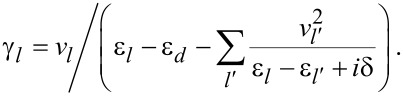


δ is a positive infinitesimal number, introduced to maintain causuality.

The operators *c_l_* and *c_r_* can be expressed in terms of the new basis as


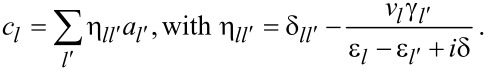


Similar expressions hold for the *r* set.

Back to Equation 1, *H_vib_* and *H_el−vib_* represent the Hamiltonians of the molecular vibrational mode and its coupling to electrons, respectively. We assume an “off-diagonal” interaction with electron hopping between local sites assisted by the vibrational mode. Assuming a local harmonic mode we write

[3]
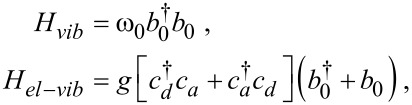


with *b*_0_ (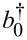) as the annihilation (creation) operator for the vibrational mode of frequency ω_0_, *g* is the coupling parameter. The Hamiltonian (Equation 1) then becomes

[4]
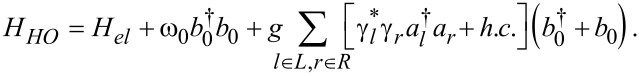


The second model considered here includes an anharmonic two-state mode. It is convenient to represent it with the Pauli matrices σ*_x,y,z_*, and to write the total Hamiltonian for the junction as

[5]
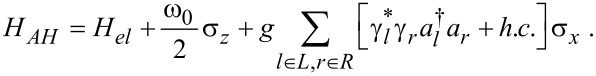


The two models, Equation 4 and Equation 5, describe electron–hole pair generation/annihilation by de-excitation/excitation of an “impurity” (vibrational mode). The left and right reservoirs defining *H_el_* in Equation 2 are characterized by a structured density of states since we had absorbed the D state in the *L* terminal, and similarly, the A level in the *R* metal. These electronic reservoirs are prepared in a thermodynamic state of temperature *T*_ν_ = 1/(*k*_B_β_ν_) and chemical potential μ_ν_, ν = *L*,*R*, set relative to the equilibrium chemical potential μ*_F_* = 0. In our description we work with 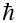 = 1 and *e* = 1. Units are revived in simulations.

## Transport

The complete information over steady-state charge and energy transport properties of molecular junctions is delivered by the so-called cumulant generating function 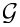, defined in terms of the characteristic function 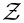 as


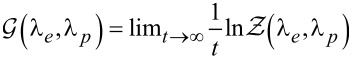


with


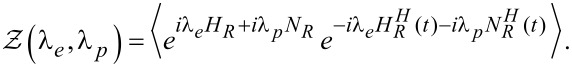


Here λ*_e_*, λ*_p_* are counting fields for energy and particles, respectively, defined for the right lead measurement, *t* is the final measurement time. The operators in this definition are 
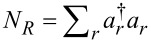
, the number operator for the total charge in the right lead, and similarly 
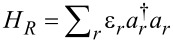
 as the total energy in the same compartment. The superscript *H* identifies the Heisenberg picture, with operators evolving with respect to the full Hamiltonian. While closed results for the CGF can be derived for junctions of noninteracting particles [38], it is challenging to calculate this function analytically for models with interactions, see for example [39]. Our simplified D–A model is one of the very few many-body models that can be solved analytically.

The CGF of the harmonic-mode junction (Equation 4) can be derived using the nonequilibrium Green’s function (NEGF) technique [40–41] assuming weak interaction between electrons and the particular vibration, employing the random phase approximation (RPA) [39,42]. This scheme involves a summation over a particular set (infinite) of diagrams (ring type) in the perturbative series, taking into account all electron scattering processes that are facilitated by the absorption or emission of a *single* quantum ω_0_. Physically, this summation collects not only sequential tunneling electrons, but all coordinated multi-tunneling processes, albeit with each electron interacting with the mode to the lowest order. The derivation of the CGF is nontrivial, and it is included in a separate communication [43]. Here we provide the final result

[6]
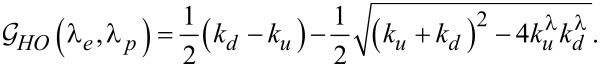


The CGF of the AH model (Equation 5) can be derived based on a counting-field dependent master equation approach [7,43],

[7]
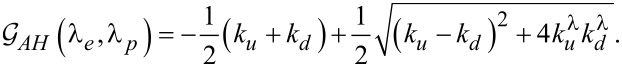


Both expressions are correct to second-order in the electron-vibration coupling *g*. It is remarkable to note on the similarity of these expressions, which were derived from separate approaches. We use the short notation λ = (λ*_p_*,λ*_e_*), where the counting fields are defined for right-lead measurements. It can be proved that our CGFs satisfy the fluctuation symmetry [44]

[8]
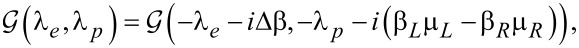


with Δβ = β*_R_*− β*_L_*. This result is not trivial: Schemes involving truncation of interaction elements may leave out terms inconsistently with the fluctuation symmetry.

Equation 6 and Equation 7 are expressed in terms of an upward (excitation) 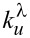 and a downward (de-excitation) 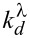 rates between vibrational states. The rates are additive in the two baths,

[9]
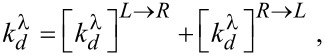


and obey the relation 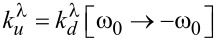. They are given by Equation 10 [7,43].

[10]
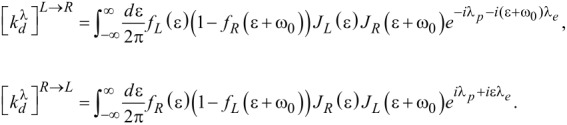


The rates *k_d_* and *k_u_* are evaluated from these expressions at λ = 0; *f*_ν_(ε) = [exp(β_ν_(ε− μ_ν_)) + 1]^−1^ is the Fermi–Dirac distribution function of the ν = *L*,*R* lead. The properties of the molecular junction are embedded within the spectral density functions, peaked around the molecular electronic energies ε*_d,a_* with the broadening Γ*_L,R_* satisfying, e.g., 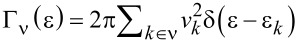,

[11]
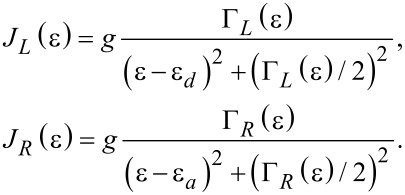


These expressions are reached through the diagonalization procedure of the electronic Hamiltonian while ignoring the real principal value term, responsible for a small energy shift of ε*_d,a_* [7]. In what follows we take Γ_ν_ as a constant independent of energy and assume broad bands with a large cutoff ±*D*, the largest energy scale in the problem.

We obtain currents and high order cumulants by taking derivatives of the CGF with respect to the counting fields. The particle 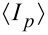 and energy 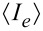 current are given by Equation 12

[12]
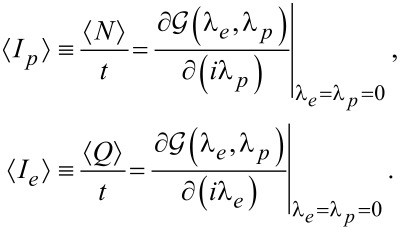


After some manipulations we reach the compact form for the harmonic (−) and anharmonic (+) models,

[13]
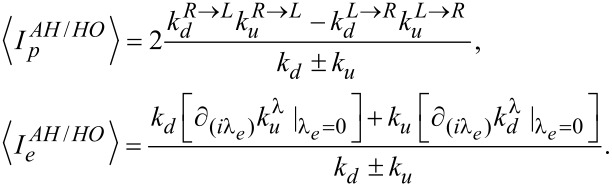


The rates are given by Equation 10 with λ = 0. It is notable that the only difference between the HO and AH models is the sign in the denominator. Note that we did not simplify the expression for the energy current 
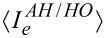
 above; the derivatives return energy transfer rates analogous to Equation 10, only with an additional energy variable in the integrand.

While figures below only display quantities related to charge and energy currents, it is useful to emphasize that the CGF contains information on fluctuations of these currents. For example, the zero-frequency noise for charge current is given from

[14]
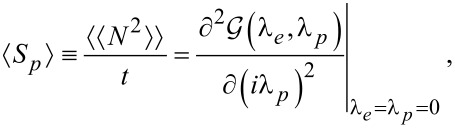


where 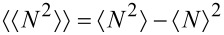 is the second cumulant.

Our derivation is based on the diagonal representation of the electronic Hamiltonian, thus the occupations of the molecular electronic states D and A follow the Fermi function by construction. In the weak coupling limit employed here, the back-action of the vibrational degrees of freedom on the electronic distribution is not included. While in other models [45] this back-action may be significant, here we argue that its role is rather small: Recent numerically exact path integral simulations [8] testify that this type of quantum master equation performs very well at weak to intermediate electron–vibration coupling, justifying our scheme. Note that in path integral simulations [8] the states D and A were absorbed into the metal leads as well, yet the electronic distribution was allowed to evolve in time, naturally incorporating the back-effect of vibrations on the electronic distribution in the steady-state limit.

## Results

We are interested in identifying signatures of mode harmonicity in transport characteristics. We set the right contact as hot, *T_R_* > *T_L_*, and write the electronic heat current extracted from the hot bath by 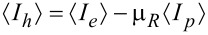. The bias is applied such that μ*_L_* > μ*_R_*, thus the macroscopic efficiency of a thermoelectric device, converting heat to work, is given by

[15]
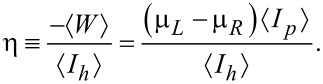


The device is operating as a thermoelectric engine when both charge and energy current flow from the hot (right) bath to the cold one. Note that according to our conventions the currents are positive when flowing from the right contact to the left.

### Linear response coefficients

In linear response, i.e., close to equilibrium, the charge current 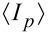 and heat current 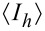 as obtained from Equation 13 can be expanded to lowest order in the bias voltage Δμ = μ*_R_*− μ*_L_* = *eV* and temperature difference Δ*T* = *T_R_*− *T_L_*. To re-introduce physical dimensions, we multiply the charge current by 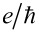 and the heat current by 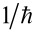. The resulting expansions are cumbersome thus we write them formally in terms of the coefficients *a_i,j_*, (*i,j* = *h,p*),

[16]
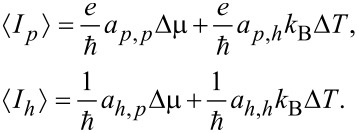


For 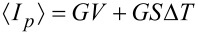 and 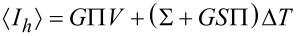 with Π being the Peltier coefficient [46–47], we identify the electrical conductance


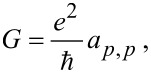


the thermopower


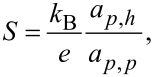


the electron contribution to the thermal conductance


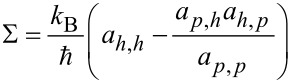


and the (dimensionless) thermoelectric figure of merit


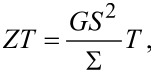


which determine the linear response thermoelectric efficiency. We obtain these coefficients numerically, by simulating Equation 13 under small biases.

**Figure 2:**
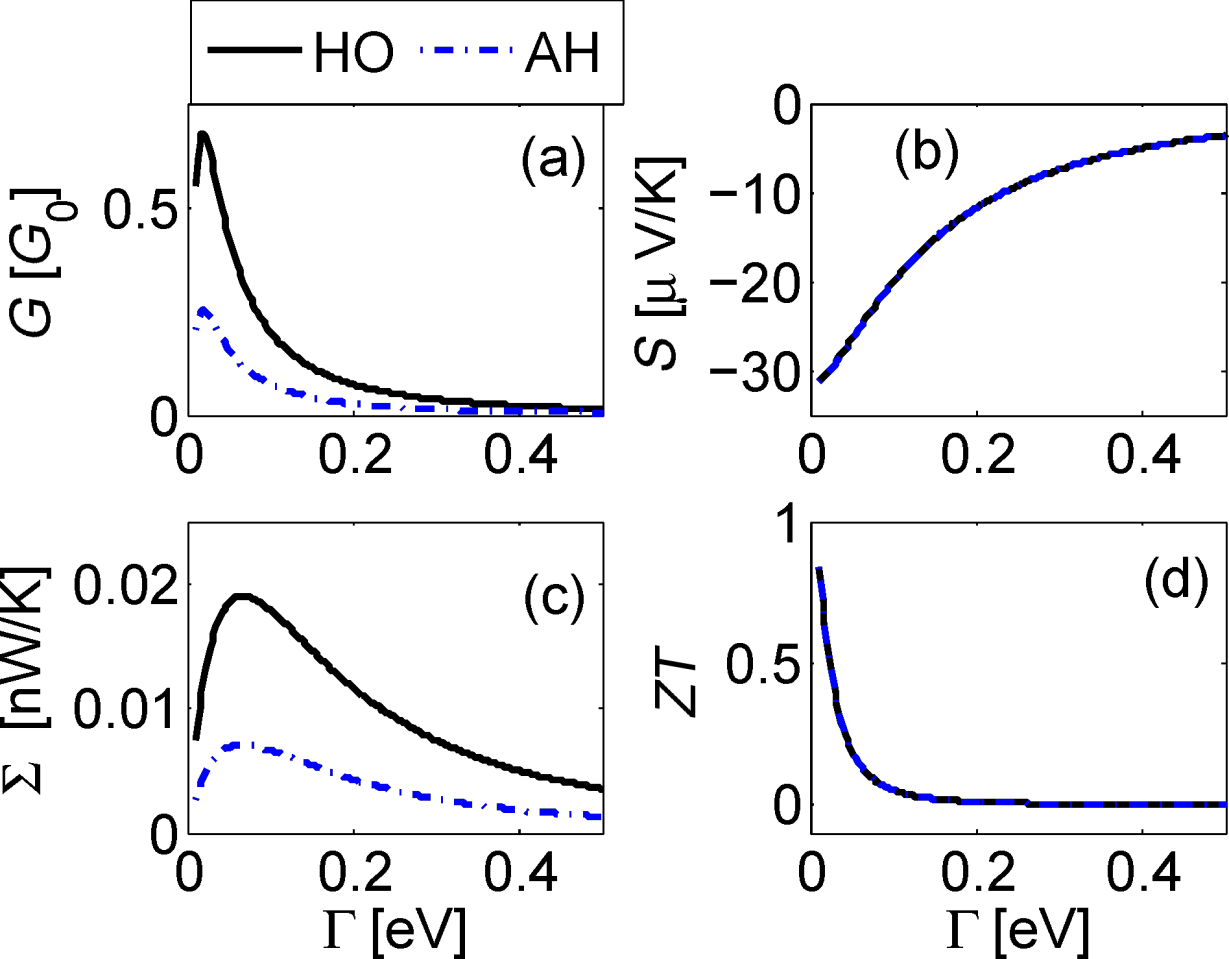
Linear response behavior of the donor–acceptor junction as a function of molecule–metal hybridization with a harmonic mode (full) and an anharmonic two-state system mode (dashed). (a) Normalized electrical conductance *G*/*G*_0_ with *G*_0_ = *e*^2^/*h*, the quantum of conductance per channel per spin. (b) Seebeck coefficient *S*. (c) Electronic thermal conductance Σ, and (d) the figure of merit *ZT*. Parameters are ε_0_ = 0.01, ω_0_ = 0.02, *g* = 0.01 in eV, room temperature *T* = 300 K. We assumed flat bands with a constant density of states.

Figure 2–Figure 4 below display the behavior of *G*, *S*, Σ and *ZT* at room temperature *T* =300 K for the harmonic and anharmonic-mode junctions. In the numerical simulations below the phononic contribution to the thermal conductance is ignored, assuming it to be small compared to its electronic counterpart. A quantitative analysis of the contribution of the phononic thermal conductance is included in the Discussion section. In addition, for simplicity, the junction is made spatially symmetric with Γ = Γ*_L,R_* and ε_0_ = ε*_d,a_*. The currents are given by Equation 13, and we make the following observations: (i) The harmonic-mode model supports higher currents relative to the two-state case, but at low temperatures, ω_0_/*T* > > 1, when the excitation rate is negligible relative to the relaxation rate, the two models provide the same results. (ii) Since the expressions for the currents in the HO and the AH models are proportional to each other, the resulting thermopower and figure of merit are identical.

Figure 2 displays transport coefficients as a function of metal–molecule hybridization assuming a resonance situation *k*_B_*T* > ε_0_. The conductances show a turnover behavior in accord with Equation 11, growing with Γ for small values Γ < ε_0_, then falling down approximately as 
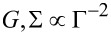
. The figure of merit shows a monotonic behavior, increasing when the broadening of levels becomes small Γ << *T* as we approach the so called “tight coupling” limit in which charge and heat currents are (optimally) proportional to each other.

**Figure 3:**
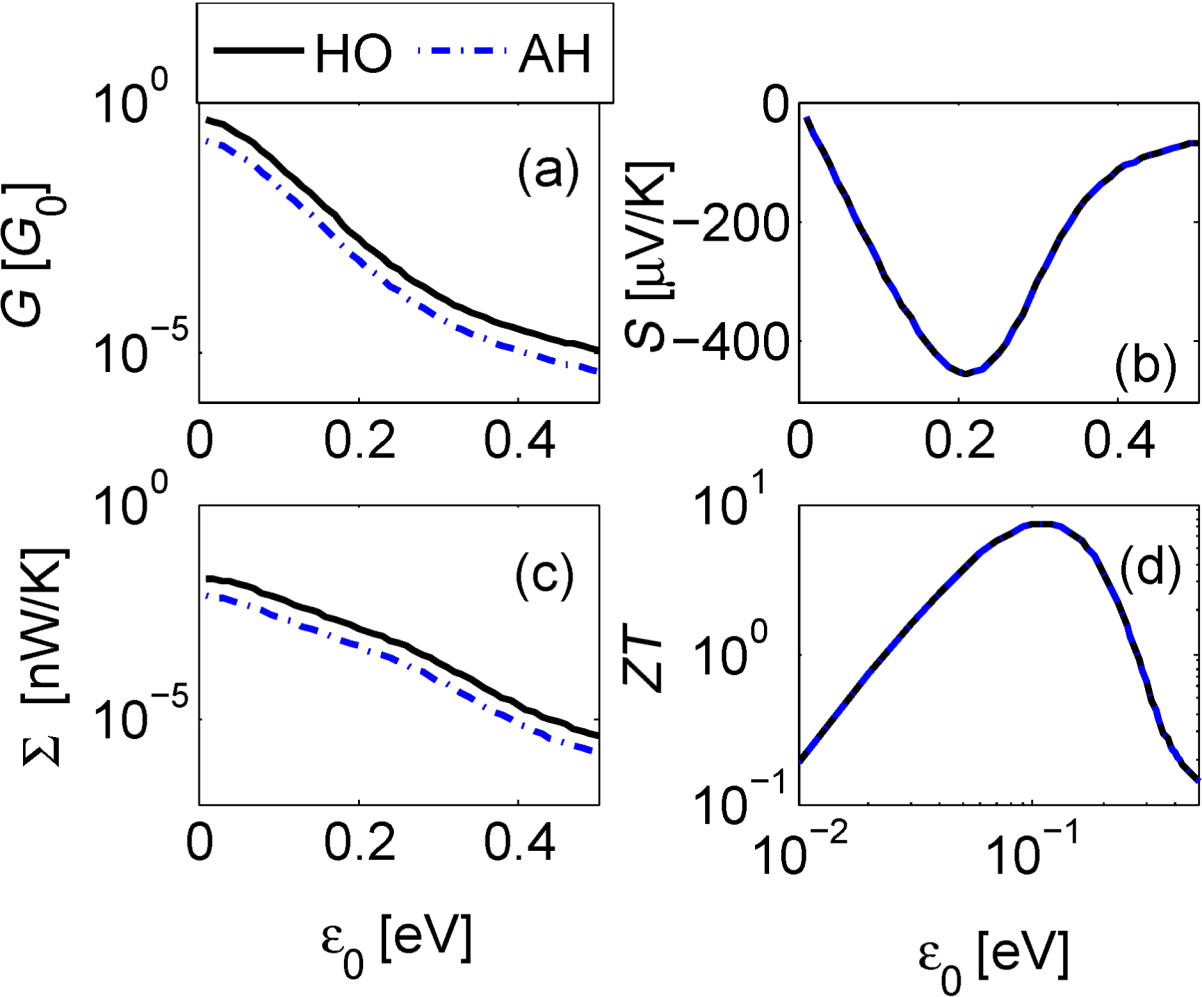
Linear response behavior of the donor–acceptor junction as a function of gate voltage. (a) Electrical conductance, (b) Seebeck coefficient, (c) electronic thermal conductance, and (d) figure of merit *ZT*. Parameters are the same as Figure 2 for Γ = 0.05 eV.

*ZT* can be significantly enhanced by tuning the molecule to an off-resonance situation, ε_0_ > *k*_B_*T*, Γ (Figure 3). We find that the electrical and thermal conductances strongly fall off with ε_0_, but the Seebeck coefficient displays a non-monotonic structure, with a maximum showing up off-resonance [48], resulting in a similar enhancement of *ZT* around ε_0_ = 0.2. It can be proven that the conductances are even functions in gate voltage, *G*(ε_0_) = *G*(−ε_0_), Σ(ε_0_) = Σ(−ε_0_) while *S*(ε_0_) = −*S*(−ε_0_), resulting in an even symmetry for *ZT* with gate voltage.

**Figure 4:**
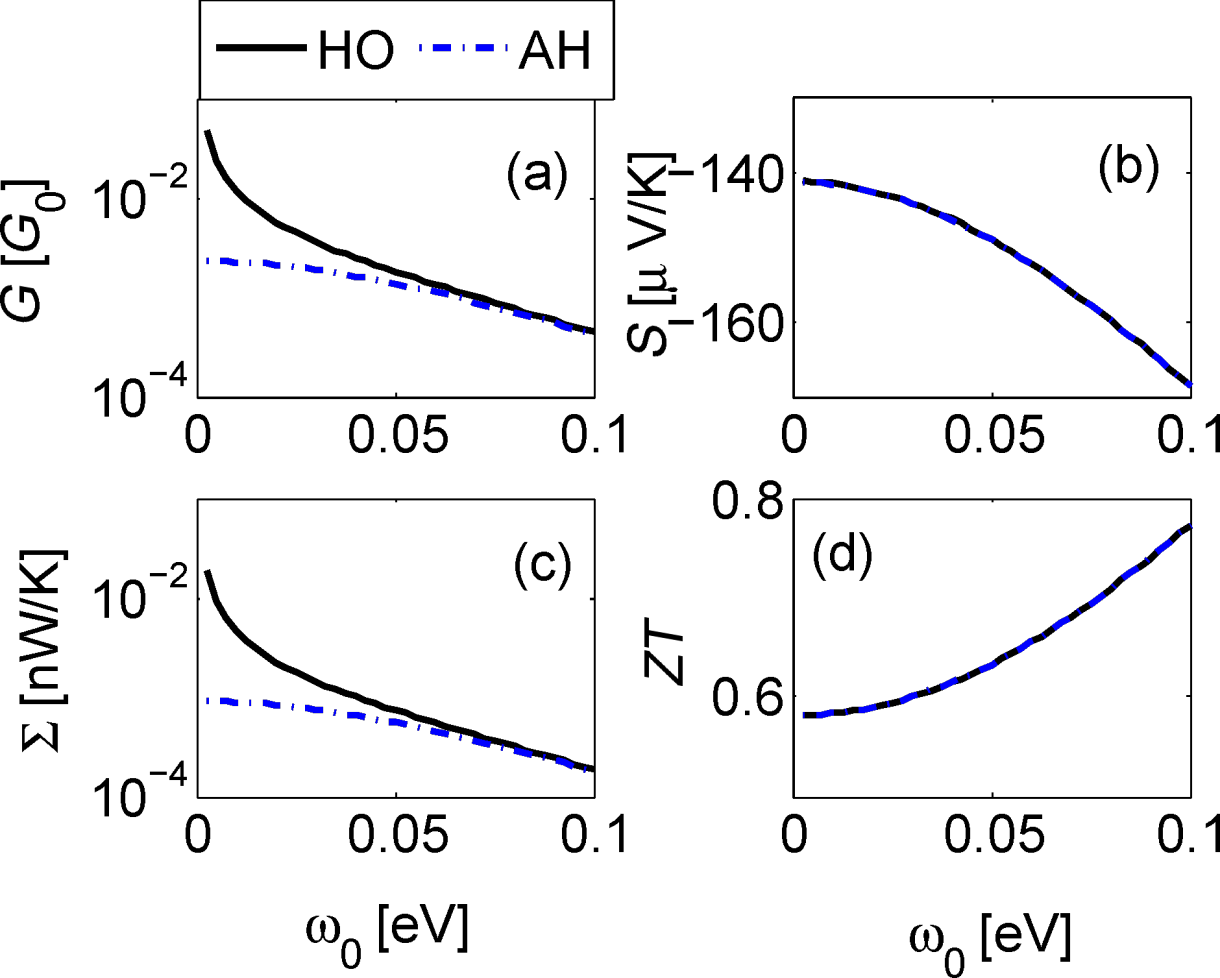
Linear response behavior of the donor–acceptor junction as a function of vibrational frequency ω_0_ for ε_0_ = 0.2 eV, Γ = 0.2 eV, *g* = 0.01 eV, and *T* = 300 K. (a) Electrical conductance, (b) Seebeck coefficient, (c) electronic thermal conductance, and (d) figure of merit *ZT*.

In Figure 4 we show transport coefficients as a function of the vibrational frequency. Parameters correspond to a resonant situation ε_0_/Γ = 1. Both *G* and Σ decay exponentially with ω_0_ when ω_0_ > *k*_B_*T*. However, the figure of merit only modestly increases with ω_0_ in the analyzed range due to the enhancement of *S* in this region. The values reported for *ZT* in Figure 4 can be increased by weakening the metal–molecule coupling energy Γ.

**Figure 5:**
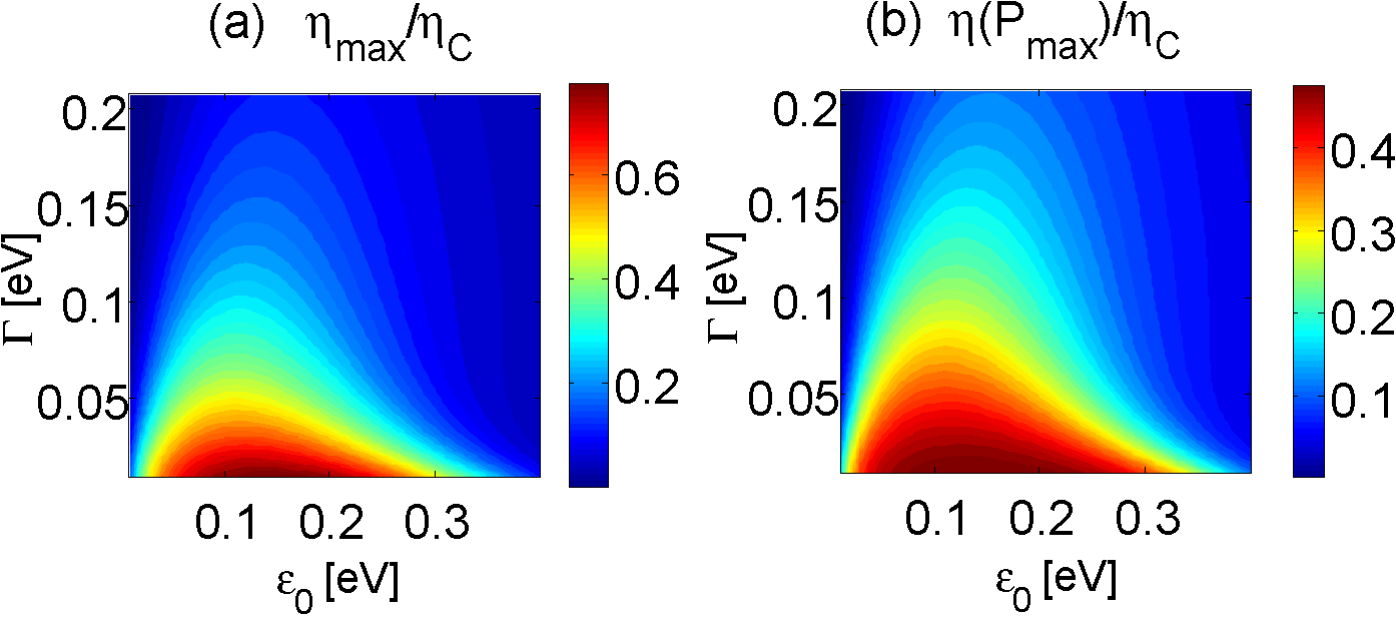
Contour plot of linear response efficiencies as a function of hybridization Γ and electronic energies (or gate voltage) ε_0_. (a) Maximum efficiency. (b) Efficiency at maximum power. Parameters are ω_0_ = 0.02 eV and *T* = 300 K.

The maximal efficiency,


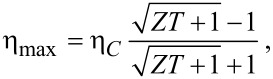


and the efficiency at maximum power [46],


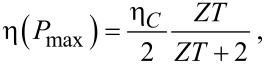


are shown in Figure 5 as a function of ε_0_ and Γ for a fixed molecular frequency ω_0_ = 0.02 eV and temperature *T* = 300 K. Here, η*_C_* = 1 − *T*_cold_/*T*_hot_ corresponds to the Carnot efficiency. By tuning the gate voltage and the molecule–lead hybridization we approach the bounds η_max_/η*_C_*→ 1, η(*P*_max_)/η*_C_*→ 1/2 [46]. Particularly, for Γ≈ 0.01 eV we obtain η_max_/η*_C_* = 0.8 at the energy ε_0_ = 0.15.

### Nonlinear performance

Nonlinear thermoelectric phenomena are anticipated to enhance thermoelectric response [49]. Elastic scattering theories of nonlinear thermoelectric transport have been developed, e.g., in [50–53], accounting for many-body effects in a phenomenological manner. Only few studies had considered this problem with explicit electron–phonon interactions, based on the Anderson–Holstein model [54] or Fermi Golden rule expressions [14].

**Figure 6:**
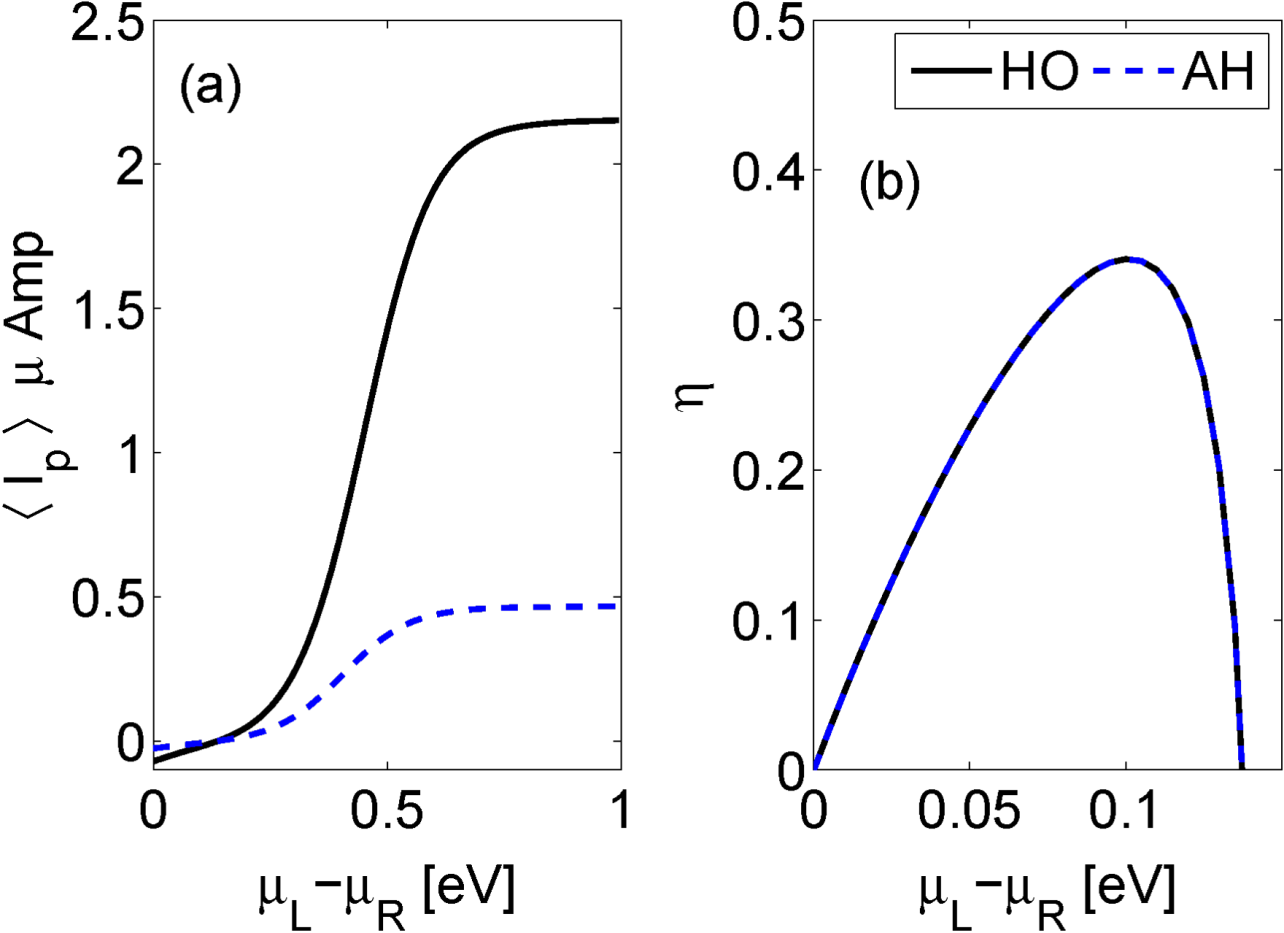
Transport beyond linear response. (a) current voltage characteristics for the harmonic (full) and anharmonic (dashed) mode models. (b) Heat to work conversion efficiency (Equation 15). Parameters are ω_0_ = 0.02, ε_0_ = 0.2, *g* = 0.01, Γ = 0.1, Γ*_ph_* = 0.002 in units of eV, and *T_L_* = 300 K, *T_R_* = 800 K and *T_ph_* = 300 K.

In Figure 6 we simulate the current–voltage characteristics and the resulting efficiency of the D–A junction beyond linear response, by directly applying Equation 13. As discussed in previous investigations [6–8], the molecular junction may break down far from equilibrium due to the development of “vibrational instability”. This over-heating effect occurs when (electron-induced) vibrational excitation rates exceed relaxation rates. To cure this physical problem, we allow the particular vibrational mode of frequency ω_0_ to relax its excess energy to a secondary phonon bath of temperature *T_ph_*. This can be done rigorously at the level of the quantum master equations and within the NEGF technique [7,43] to yield the rates


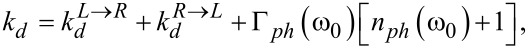



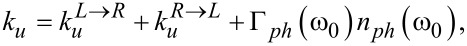


with 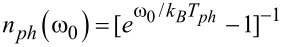 and a damping term Γ*_ph_*(ω_0_). Interestingly, we confirmed (not shown) that this additional energy relaxation process does not modify the thermoelectric efficiency displayed in Figure 6b.

## Discussion and Prospect

We focused on two-site electronic junctions in which electron transfer between sites is assisted by a particular mode, harmonic or anharmonic (two-state system). The complete information over steady state transport behavior is catered by the cumulant generating function, which we provide here for the HO and the AH mode models, valid under the approximation of weak electron–vibration interaction. We explored linear-response properties, the electrical and thermal conductances *G* and Σ, as well as the Seebeck coefficient *S*, the thermoelectric figure of merit *ZT*, and the resulting efficiency. We further examined current–voltage behavior and the heat-to-work conversion efficiency far-from-equilibrium. We found that *G* and Σ (more generally, the charge and energy currents) are sensitive to the properties of the mode, while *S* and *ZT* are insensitive to whether we work with a harmonic mode or a truncated two-state model.

Several comments are now in place:

(i) **Genuine anharmonicity.** We examined the role of mode anharmonicity by devising a two-state impurity model. It should be emphasized that in the context of molecules, the two-state impurity does not well represent vibrational anharmonicity at high temperatures, as many states should then contribute. Furthermore, it misses an explicit parameter tuning the potential anharmonicity. However, the AH model allows for a first indication on how deviations from harmonicity reflect in transport behavior. The HO and AH models have similar CGFs, yielding currents which are proportional, thus an identical thermoelectric efficiency. It can be readily shown that an *n*-state truncated HO provides a figure of merit identical to the infinite-level HO model, but it is interesting to perform more realistic calculations and consider, e.g., a morse potential to represent a physical anharmonic molecular vibration. In this case, an analytical form for the CGF is missing, but one could still derive the charge current directly from a quantum master equation formalism, to obtain the performance of the system. We expect that with a genuine anharmonic potential, *S* and *ZT* would show deviations from the harmonic limit, as different pathways for transport open up. Overall, we believe that our results here indicate on the minor role played by mode anharmonicity in determining heat-to-work conversion efficiency.

(ii) **Direct tunneling.** Our analysis was performed while neglecting direct electron tunneling between the D and A sites. This effect could be approximately re-instituted by assuming that coherent transport proceeds in parallel to phonon-assisted conduction, accounting for the coherent contribution using a Landauer expression, see, e.g., [3,14]. Indeed, path integral simulations indicted that in the D–A model, coherent and the incoherent contributions are approximately additive [8].

(iii) **Strong electron-phonon interaction.** The CGFs (Equation 6 and Equation 7) are exact to all orders in the metal–molecule hybridization but perturbative (to the lowest nontrivial order) in the electron phonon coupling *g*. This is evident from the structure of the rate constants in Equation 10, as electron transfer is facilitated by the absorption/emission of a single quantum ω_0_. In numerical simulations we typically employed *g* = 0.01 eV and ω_0_ = 0.02 eV. This value for *g* may seem large given the perturbative nature of our treatment requiring *g*/ω_0_ << 1. However, since in the present weak-coupling limit the current simply scales as *g*^2^, Equation 13, our simulations in this work are representative, and can be immediately translated for other values for *g*. It is of interest to generalize our results and study the performance of the junction with strong electron–phonon interaction, e.g., by using a polaronic transformation [55–59].

(iv) **Phononic thermal conductance.** We studied here electron transfer through molecular junctions, but did not discuss phonon transport characteristics across the junction, mediated by molecular vibrational modes. Consideration of the phononic thermal conductance κ*_ph_* is particularly important for a reliable estimate of *ZT*, as the thermal conductance Σ should include contributions from both electrons and phonons. We now estimate κ*_ph_*. The quantum of thermal conductance, an upper bound for ballistic conduction, is given by 
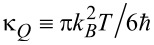
[60]. At room temperature, this yields κ*_Q_* = 0.28 nW/K, which exceeds the electronic thermal conductance obtained in our simulations, to dominate the total thermal conductance and predict (significantly) lower values for the figure of merit. However, one should recognize that at high temperatures the ballistic bound for phonon thermal conductance is far from being saturated as was recently demonstrated in [61]. In particular, the phononic thermal conductance of a two-level junction was evaluated exactly in [62], and it significantly falls below the harmonic bound [61].

For a concrete estimate, we adopt the perturbative (weak mode-thermal bath) expression for the phononic current through a two-state junction developed in [63] and further examined in [64],

[17]
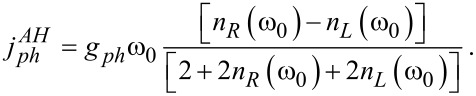


Here, *g_ph_* is the interaction strength of the local vibrational mode to the phononic environments at the two terminals (assuming identical interaction strengths). The baths are characterized by their Bose–Einstein distribution functions *n*_ν_(ω). In the case of a local harmonic mode, Equation 17 holds, only missing its denominator. Using ω_0_ = 0.02 eV, we receive an estimate for the phononic thermal conductance 
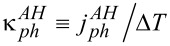
, 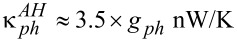. Thus, as long as the mode-bath coupling *g_ph_* is taken as weak, for example, *g_ph_* <5 meV for the data of Figure 2, the electronic contribution to the thermal conductance dominates the total thermal conductance and our simulations are intact. Similar considerations hold for harmonic-mode junctions. Proposals to reduce the coherent phononic thermal conductance by quantum interference effects [65] and through-space designs [66] could be further considered.

(v) **High order cumulants.** The cumulant generating functions, Equation 6 and Equation 7, contain significant information. For example, one could examine the (zero-frequency) current noise, to find out to what extent it can reveal microscopic molecular information.

(vi) **Methodology development.** The cumulant generating function of the HO model was derived from an NEGF approach [43]. The corresponding function for the AH model was reached from a master equation calculation [7,43]. Both treatments are perturbative to second order in the electron–vibration interaction. We take into account all electron scattering processes that are facilitated by the absorption or emission of a *single* quantum ω_0_. It is yet surprising to note on the direct correspondence between NEGF and master equation results, as derivations proceeded on completely different lines. In particular, the NEGF approach was done at the level of the RPA approximation to guarantee the validity of the fluctuation theorem. The master equation approach has been employed before to study currents (first cumulants) in the HO model [7], showing exact agreement with NEGF expressions presented here. This agreement, as well as supporting path integral simulations [8], indicate on the accuracy and consistency of the master equation in the present model. Given its simplicity and transparency, it is of interest to extend this method and examine higher order processes in perturbation theory, to gain further insight on the role of electron–vibration interaction in molecular conduction.

(vii) **Efficiency fluctuations.** We focused here only on averaged-macroscopic quantities. However, in small systems fluctuations in input heat and output power are significant, resulting in “second law violations” as predicted from the fluctuation theorem [44], to, e.g., grant efficiencies exceeding the thermodynamic bound. To analyze the distribution of efficiency, the concept of “stochastic efficiency” has been recently coined and examined [67–68]. In a separate contribution [43] we extend the present analysis and describe the characteristics of the stochastic efficiency in our model, particularly, we explore signatures of mode anharmonicity in the statistics of efficiency.

(viii) **Molecular calculations.** It is of interest to employ our expressions and examine heat-to-work conversion efficiency in realistic molecular junctions. Our results demonstrate that the conversion efficiency can be improved by working in the off-resonant limit, Γ/ε_0_ << 1, as well, when tuning ε_0_ through a gate voltage to ε_0_/*k*_B_*T*≈ 5. In such situations, the figure of merit *ZT* can be made large since one can tune *S* to large values (though the conductances are small). This suggests that in the D–A class of molecules one should focus on enhancing the thermopower as a promising mean for making an overall improvement in efficiency.

In our ongoing work we are pursuing some of these topics. The derivation of the CGFs employed here and the behavior of efficiency fluctuations in linear response, and beyond that, are detailed in [43]. In [14] we derive thermoelectric transport coefficients for the dissipative D–A model, beyond linear response, and describe the operation of thermoelectric diodes and transistors.
